# Drug Metabolism in Human Brain: High Levels of Cytochrome P4503A43 in Brain and Metabolism of Anti-Anxiety Drug Alprazolam to Its Active Metabolite

**DOI:** 10.1371/journal.pone.0002337

**Published:** 2008-06-11

**Authors:** Varsha Agarwal, Reddy P. Kommaddi, Khader Valli, Daniel Ryder, Thomas M. Hyde, Joel E. Kleinman, Henry W. Strobel, Vijayalakshmi Ravindranath

**Affiliations:** 1 Division of Molecular and Cellular Neuroscience, National Brain Research Centre, Nainwal Mode, Manesar, India; 2 Department of Biochemistry and Molecular Biology, University of Texas Medical School, Houston, Texas, United States of America; 3 Clinical Brain Disorders Branch, Section on Neuropathology, Division of Intramural Research Programs (DIRP)/National Institute of Mental Health (NIMH)/National Institutes of Health (NIH), Bethesda, Maryland, United States of America; University of Massachusetts Medical School, United States of America

## Abstract

Cytochrome P450 (P450) is a super-family of drug metabolizing enzymes. P450 enzymes have dual function; they can metabolize drugs to pharmacologically inactive metabolites facilitating their excretion or biotransform them to pharmacologically active metabolites which may have longer half-life than the parent drug. The variable pharmacological response to psychoactive drugs typically seen in population groups is often not accountable by considering dissimilarities in hepatic metabolism. Metabolism in brain specific nuclei may play a role in pharmacological modulation of drugs acting on the CNS and help explain some of the diverse response to these drugs seen in patient population. P450 enzymes are also present in brain where drug metabolism can take place and modify therapeutic action of drugs at the site of action. We have earlier demonstrated an intrinsic difference in the biotransformation of alprazolam (ALP) in brain and liver, relatively more α-hydroxy alprazolam (α-OHALP) is formed in brain as compared to liver. In the present study we show that recombinant CYP3A43 metabolizes ALP to both α-OHALP and 4-hydroxy alprazolam (4-OHALP) while CYP3A4 metabolizes ALP predominantly to its inactive metabolite, 4-OHALP. The expression of CYP3A43 mRNA in human brain samples correlates with formation of relatively higher levels of α-OH ALP indicating that individuals who express higher levels of CYP3A43 in the brain would generate larger amounts of α-OHALP. Further, the expression of CYP3A43 was relatively higher in brain as compared to liver across different ethnic populations. Since CYP3A enzymes play a prominent role in the metabolism of drugs, the higher expression of CYP3A43 would generate metabolite profile of drugs differentially in human brain and thus impact the pharmacodynamics of psychoactive drugs at the site of action.

## Introduction

Cytochromes P450 (P450), the major class of drug metabolizing enzymes, play an important role in determining therapeutic efficacy through the conversion of drugs to their active metabolites as well as detoxification to more hydrophilic metabolites that facilitate rapid excretion [1]. Although liver is the major organ that contributes to P450-mediated drug metabolism, biotransformation at the site of action such as the brain by extrahepatic P450 enzymes is also an important determinant of therapeutic outcome [2]. Previous studies from our laboratory and others have shown that brain P450 enzymes from rodents [3],[4] and human brain [5],[6] metabolize a variety of xenobiotics including drugs. Further, the preferential localization of xenobiotic metabolizing enzymes within specific cell types in these organs provides such cells remarkable capability to metabolize foreign compounds including drugs [1],[2],[7]. Metabolism of psychoactive drugs directly in the brain may lead to local pharmacological modulation at the site of action and result in variable drug responses [7]. The focus of our current study is the human CYP3A family members that contribute significantly to drug metabolism. Some of the important substrates of CYP3A4 include macrolide antibiotics, anti-arrhythmics, antidepressants, anxiolytics including alprazolam, midazolam and diazepam [8], immune system modulators and an array of calcium channel blockers. CYP3A4 is also involved in the metabolism of endogenous steroidal substrates including testosterone, progesterone, and androstenedione [9].

The human CYP3A subfamily consists of four functional genes, CYP3A4, CYP3A5, CYP3A7, CYP3A43, and two pseudogenes, CYP3A5P1 and CYP3A5P2. These genes are located in tandem on chromosome 7 [10]. Expression of CYP3A genes is both tissue and cell specific and exhibits inter-individual variation [11]. Of all CYP3A subfamily members, CYP3A4 accounts for 60% of total P450 content of adult human liver and metabolizes almost 50% of the therapeutic drugs known [12]. CYP3A5 expression is one-fifth of CYP3A4 levels in liver [13] although it has been reported to be higher in African American population [14]. CYP3A7 is predominately a fetal enzyme [15]. CYP3A43 is a recently discovered member of this family [10],[16]. It is expressed at very low levels in liver, prostrate and testis and therefore has been considered not to play a significant role in drug metabolism [17].

Alprazolam, a triazolobenzodiazepine, is an anti-anxiety drug and one of the most widely prescribed benzodiazepines used in the treatment of anxiety and panic disorders. In human liver alprazolam is metabolized to hydroxylated metabolites by CYP3A enzymes [18]. α-Hydroxy alprazolam (α-OHALP) is the minor but pharmacologically active metabolite [19], while 4-hydroxy alprazolam (4-OHALP) is the major and pharmacologically less active metabolite [20]. ALP is also metabolized to its hydroxylated metabolites by both rat and human brain microsomes indicating in situ metabolism in the target organ. There is an intrinsic difference in the biotransformation of alprazolam in brain and liver. Although the presence of P450 enzymes in brain is one-tenth to one-fifteen of liver [4], the metabolism of alprazolam by rat and human brain microsomes to the active metabolite, (α-OHALP) is relatively high [4]. Further, the amount of 4-hydroxy alprazolam formed in rat brain is 3.2% of the concentration in liver, α-hydroxy alprazolam concentration is 75% of the corresponding level in liver [4]. We have now examined this biotransformation in greater detail by studying the metabolism of alprazolam by individual members of the CYP3A family; namely CYP3A4 and CYP3A43, a relatively less studied enzyme, generated by recombination. We have further quantitated the expression of the CYP3A genes in human brain using quantitative real time PCR and examined the correlation with the metabolites formed in human brain microsomes.

## Results

### Constitutive Expression of CYP3A43 in human brain

**Figure 1:**
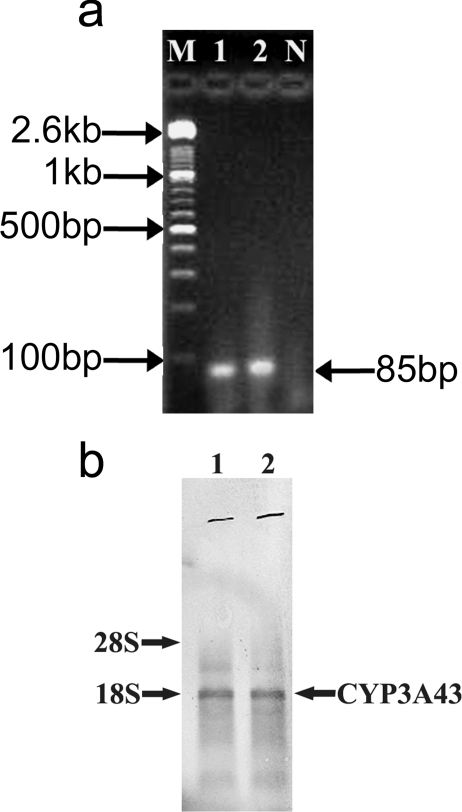
Constitutive expression of CYP3A43 mRNA in human brain cortex. (a) Total RNA was extracted from human brain cortex of 2 subjects and the 3′-UTR of CYP3A43 was amplified by RT-PCR (lanes 1 and 2). Lane N denoted reaction performed without template DNA. The size of the PCR product was 85bp. (b) Total RNA from human brain cortex was electrophoresed, transferred to positively charged nylon membrane and hybridized with antisense riboprobe prepared using the partial cDNA to CYP3A43. The mobility of 18S and 28S ribosomal RNA on the agarose gel is indicated.

**Figure 2:**
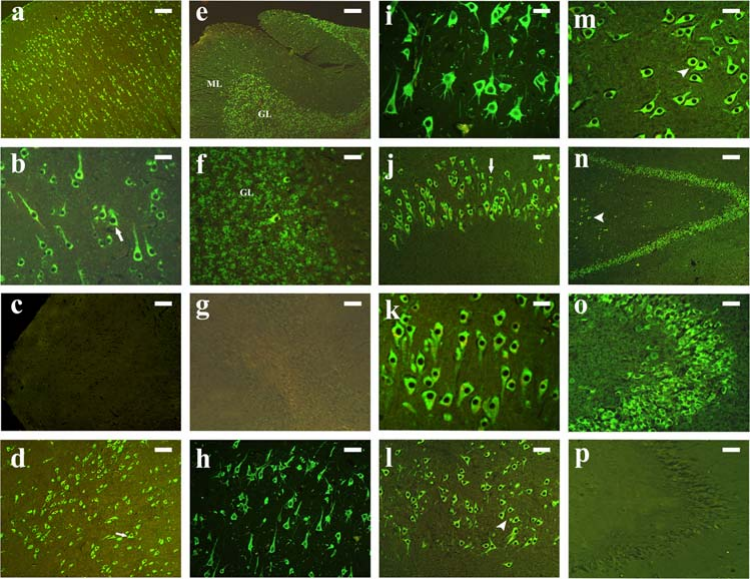
Localization of *CYP3A43* mRNA in human brain by fluorescence in situ hybridization. (a) Intense fluorescence was seen in neuronal cell layers of frontal cortex. Differential localization was seen delineating the laminar architecture of the cortex. Bar = 100 µm (b) Higher magnification of cortical neurons (arrow). Bar = 25 µm (c) Control section hybridized with sense probe did not show any fluorescence. Bar = 100 µm (d) Reticular neurons in the midbrain expressed CYP3A43 mRNA. Bar = 100 µm (e) Fluorescent labelling of the granule cell layer (GL) in human cerebellum. Sparse staining was observed in the molecular layer (ML). Bar = 100 µm (f) Higher magnification of neurons of the granule cell layer in cerebellum. Bar = 25 µm (g) Control section hybridised with sense probe. Bar = 100 µm (h) CYP3A43 expression was seen in pyramidal neurons of CA1 in the hippocampus. Bar = 50 µm (i) Higher magnification of CA1 neurons. Bar = 25 µm (j) Robust staining of the CA2 pyramidal cell layer (arrow) of the hippocampus. Bar = 50 µm (k) Higher magnification of pyramidal neurons in the CA2 subfield. Bar = 25 µm (l) Intense fluorescence was seen in the CA3 pyramidal neurons (arrow head) of the human brain. Bar = 50 µm (m) Higher magnification of CA3 neurons (arrow head). Bar = 25 µm (n) Intense fluorescence was observed in the dentate gyrus. Staining of the interneurons of the hilus (arrow head) was also observed. Bar = 100 µm (o) Higher magnification of the granule cells in the dentate gyrus. Bar = 25 µm (p) Control section of hippocampus hybridized with sense probe did not show any fluorescence. Bar = 100 µm

Using RT-PCR, we amplified 85bp spanning partial exons 12–13 of the 3′ UTR of CYP3A43 such that there was no identity with any other member of CYP3A subfamily using RNA from human brain cortex (Fig 1a). This amplicon was cloned and used as riboprobe for northern blot and localization using FISH. Northern blot analysis using the antisense riboprobe revealed the expression of CYP3A43 mRNA in human brain (Fig 1b). The molecular mass of the transcript was approximately 1.6 Kb, which is similar to that seen for hepatic CYP3A43. No signal was observed, when the northern blot was hybridized with sense probe (data not shown). Localization of constitutively expressed CYP3A43 mRNA in human brain using fluorescence in situ hybridization clearly demonstrates the differential expression of CYP3A43 mRNA in different regions of human brain. CYP3A43 expression was localized in cortical neurons (Fig 2a and b), pyramidal neurons of the hippocampus (Fig 2h–m), granule cell layers of the dentate gyrus (Fig 2n and o), and reticular neurons of the midbrain (Fig 2d). In the cerebellum, granule cell layer showed intense fluorescent labeling (Fig 2e); occasional staining was seen in the molecular layer (Fig 2f), however, Purkinje cells showed no fluorescence (Fig 2f).

### Relative expression of CYP3A4 and CYP3A43 in human brain

**Figure 3:**
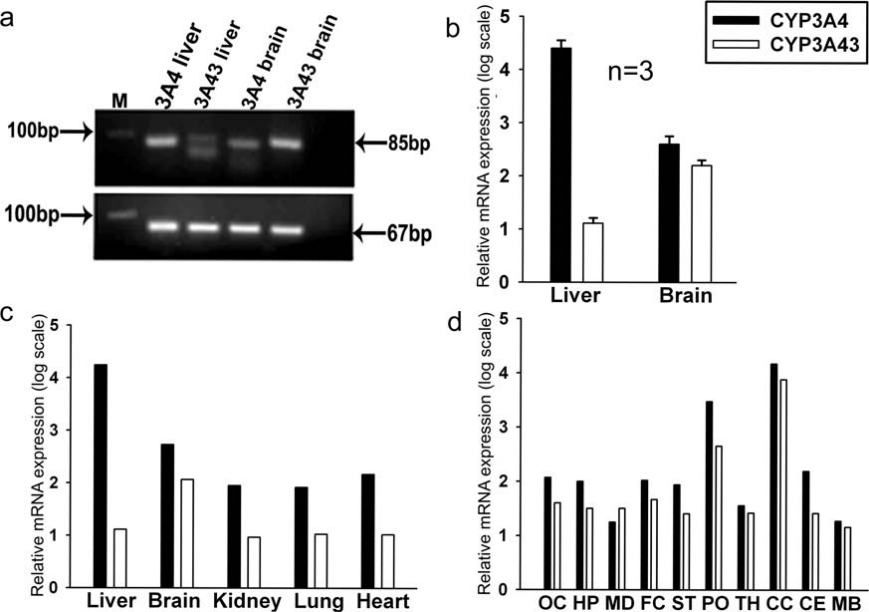
Quantitative assessment of the expression of CYP3A4 and CYP3A43 in human brain cortex. (a) RT-PCR amplification of the 85bp of 3′-UTR of CYP3A43 and 78bp of 3′-UTR of CYP3A4 using human brain and liver RNA. (b) qRT-PCR analysis using human liver and brain RNA (n = 3) showed the relative higher expression of CYP3A43 in brain compared to liver from the same individuals. Quantitation of the relative expression of CYP3A4 and CYP3A43 in (c) different human brain tissues (n = 1) and (d) from different regions of the brain from same individual (n = 3).

**Figure 4:**
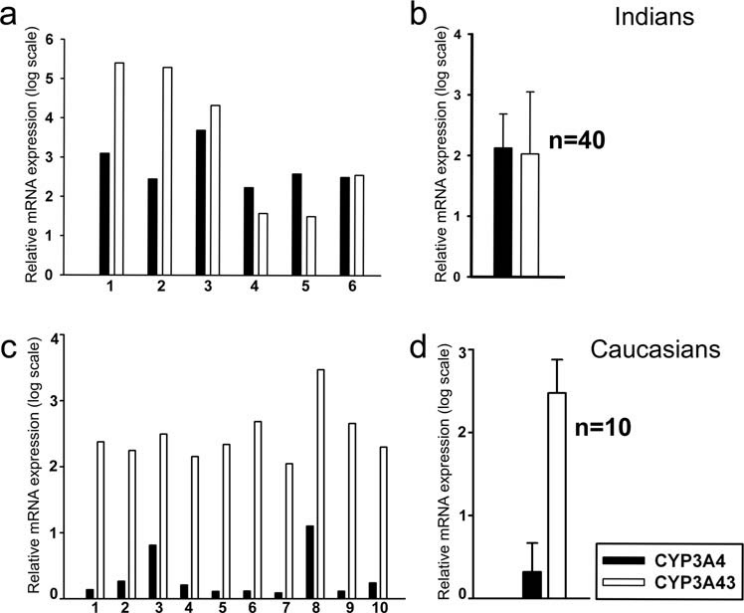
Expression of CYP3A43 and CYP3A4 in human brain samples from different population groups. Expression of CYP3A43 and CYP3A4 in 6 individual samples of human brain cortex obtained from Indians depict the inter-individual variation (a). Expression of CYP3A43 and CYP3A4 averaged across 40 samples of human brain cortex from India (b). Comparison of CYP3A4 and CYP3A43 transcripts among 10 Caucasian brain samples (c) demonstrated that on average (d) CYP3A43 expression is 170 fold higher than CYP3A4. All samples were normalized using 18S rRNA.

CYP3A43 is expressed at relatively low levels in liver as compared to CYP3A4, expression of CYP3A43 mRNA was about 0.1% of CYP3A4 in human liver samples (Fig 3b) while in the brain CYP3A43 expression is relatively higher (Fig 3a). This was confirmed by quantitative real time PCR (Fig 3b). Expression of CYP3A4 and CYP3A43 in different tissues obtained at autopsy from the same individuals showed that CYP3A43 was highest in brain as compared to liver, kidney, lung, and heart (Fig 3c). Within the CNS, CYP3A43 expression was highest in the pons and cervical cord (Fig 3d). Out of the 40 samples of human brain cortex obtained at autopsy from subjects of Indian origin that were analyzed, 3 samples had 10–100 fold higher expression of CYP3A43 compared to CYP3A4 while 2 showed a10 fold higher degree of expression of CYP3A4, and 35 samples expressed both the genes in equal amounts (Fig 4a and b). Amongst the 10 caucasian samples, CYP3A43 transcript levels were, on average, 170 fold higher than CYP3A4 (Fig 4c and d).

### Cloning and expression of CYP3A4 and CYP3A43

**Figure 5:**
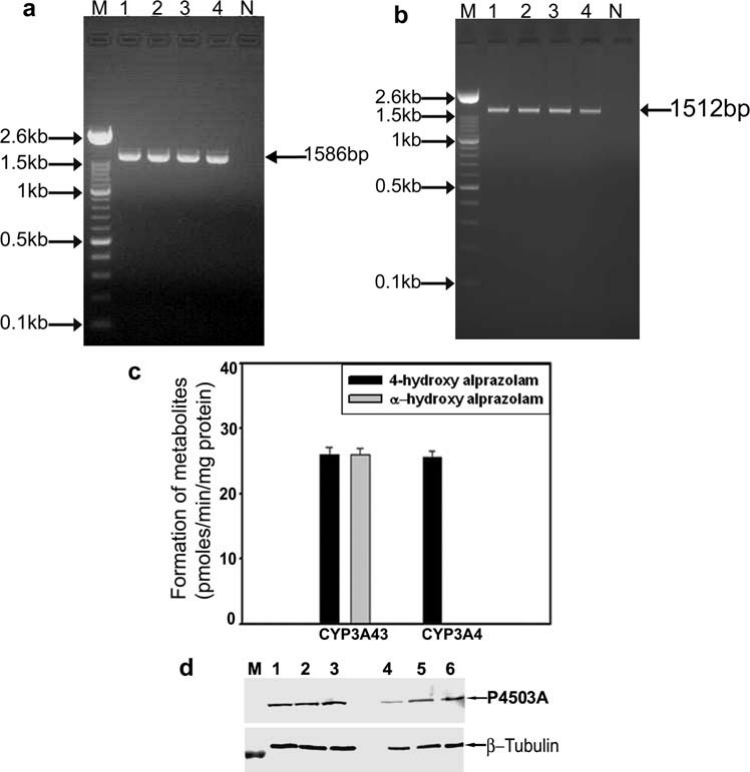
Cloning and expression CYP3A43 and CYP3A4 and metabolism of alprazolam, in vitro by recombinant enzymes. The complete open reading frame of CYP3A43 and CYP3A4 were amplified by RT-PCR using total RNA from four autopsy human brain samples. The amplicons of 1586bp and 1512bp were generated for CYP3A4 and CYP3A43, respectively (a, and b, from lanes 1–4). M represents 100bp ladder and Ne, the control reaction performed without template DNA. The amplicons were cloned into pcDNA 3.1 for expression in COS-1 cells. (c) Recombinant CYP3A43 metabolized alprazolam to α-OHALP and 4-OHALP in similar amounts (26 pmoles/min/mg protein), while recombinant CYP3A4 metabolized alprazolam only to 4-OHALP. The values are mean ISD (n = 3 independent experiments). (d) Immunoblot of recombinant CYP3A43 and CYP3A4 normalized using β-tubulin.

The complete ORF of CYP3A4 and CYP3A43 were amplified by RT-PCR using total RNA from human brain cortex (Fig 5a and b) and cloned into mammalian expression vectors for expression in COS-1 cells. CYP3A43 metabolized alprazolam equally to both α-OHALP and 4-OHALP. CYP3A4, on the other hand metabolized alprazolam principally to 4-OHALP (Fig 5c). No metabolite of alprazolam was detected in cells transfected with vector alone or vector containing the cDNA in reverse orientation (data not shown). Western blotting confirmed the expression of full-length recombinant CYP3A4 and CYP3A43 in COS-1 cells (Fig 5d).

### Metabolism of alprazolam by human brain

**Figure 6:**
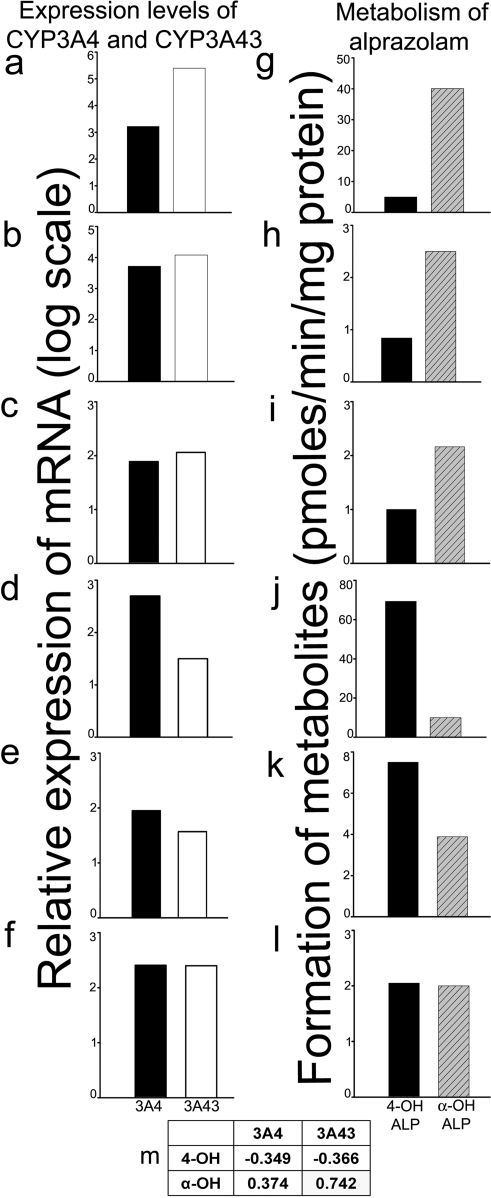
Metabolism of alprazolam to α-OHALP and 4-OHALP by human brain, in vitro. The expression levels of CYP3A4 and CYP3A43 were compared with the rates of formation of α-OHALP and 4-OHALP in samples of cortex obtained from 6 human brain subjects. Subjects represented in (a, b, c) expressed high levels of CYP3A43 and relatively higher amounts of α-OHALP was formed compared to 4-OHALP (g, h, i). Subjects (d, e) expressed high levels of CYP3A4 and formed relatively higher amounts of 4-OHALP (j, k). Subject (f) showed similar levels of expression CYP3A4 and CYP3A43 which was reflected in the amount of hydroxylated metabolite formed. Pearson correlation analysis showed high degree of correlation between the expression levels of CYP3A43 and amounts of α-OHALP formed (m; r = 0.74).

Total membrane preparations from human brain cortex metabolized alprazolam to its hydroxylated metabolites. Human brain samples that expressed higher levels of CYP3A43 metabolized alprazolam to higher amounts of α-OHALP (Fig 6 a, g; b, h; c, i) whereas, those that expressed higher levels of CYP3A4 formed more 4-OHALP (Fig 6 d, j; e, k). In addition, brain samples that expressed both CYP3A4 and CYP3A43 in equal amounts metabolized alprazolam to both α-OHALP and 4-OHALP (Fig 6 f, l). Pearson analysis showed high degree correlation between expression levels of CYP3A43 and the formation of α-OHALP (Fig 6m).

### Relative expression of CYP3A family in human brain

**Figure 7:**
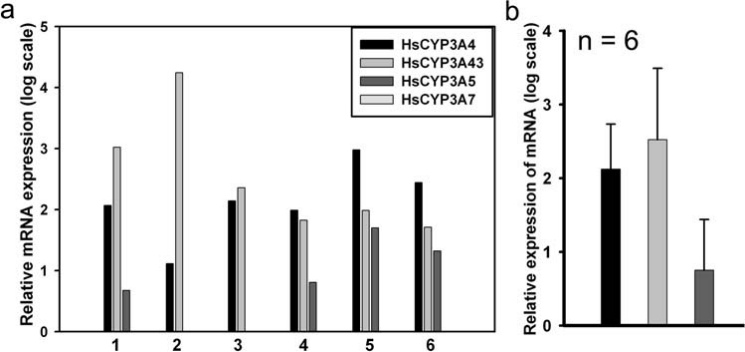
Relative expression of functional genes of CYP3A subfamily members in human brain samples. (a) The levels of CYP3A4, CYP3A5, CYP3A7, and CYP3A43 mRNA were quantified by qRT-PCR in the 6 human brain subjects. The expression of CYP3A5 was significantly lower then that of CYP3A4 and CYP3A43 and was detected only in four out of six samples. CYP3A7 was not detected in any of the subjects. (b) The average of the six samples analyzed.

The expression of both CYP3A4 and CYP3A43 were higher in 6 brain samples examined in comparison to CYP3A5, which was detected only in four samples. CYP3A7, a fetal enzyme was not detected in any of the sample analyzed (Fig 7a and b).

### Splice variants of CYP3A43 and CYP3A4 in human brain

**Figure 8:**
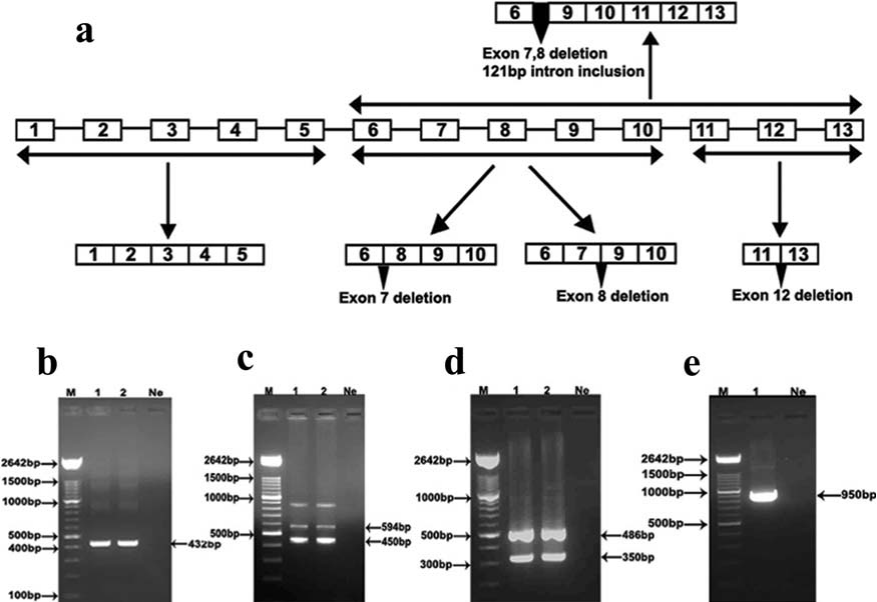
Amplification of exonic regions of the ORF of CYP3A43 and CYP3A4 mRNA by RT-PCR. (a) The schematic representation of 13 exons of CYP3A43 along with their splice variants detected by RT-PCR is depicted. (b) A 432bp amplicon was amplified spanning the exons 1–5 (lanes 1 and 2). (c) Two amplicons (594bp and 450bp) were generated when exons 6–10 were amplified (lanes 1 and 2). The 594bp product was the expected amplicon while the other amplicon (450bp) was a mixture of two PCR products of which one had a deletion of exon 7 and other had a deletion of exon 8. (d) Amplification of the region spanning exons 11–13 gave two amplicons of sizes 486bp and 350bp. The 486bp product was the expected size while 350bp product represented a splice variant that had the deletion of exon 12. (e) Amplification of exons 6–13 generated an amplicon (950bp) representing a splice variant having deletion of both exon 7 and 8 and partial inclusion of 121bp intron 7.

CYP3A43 has 13 exons. We amplified the exonic regions 1–5, 6–10, 11–13, and 6–13 (Fig 8a). The primers were designed on the exon-exon boundaries such that intron inclusion in any splice variant would be detected. RT-PCR amplification of exons 1–5 generated a product of the expected size (432bp; Fig 8b), however, amplification of exons 6–10 generated two splice variants having deletion of exon 7 (149bp) or exon 8 (128bp, Fig 8c). Both these splice variants would potentially result in pre-mature stop codon. Amplification of exons 11–13 generated a product with deletion of exon 12 (126bp, Fig 8d). Finally we amplified exons 6–13 of CYP3A43 and found a splice variant having deletion of exons 7 and 8 and partial inclusion of 121bp of intron 7. This variant would potentially translate to a functional protein (Fig 8e). Similar amplifications of the ORF of CYP3A4 did not yield any splice variants (data not shown).

## Discussion

Differential expression of CYP enzymes in organ and cell-specific manner would contribute greatly to the pharmacodynamics of drug action in the target organ. The metabolism of alprazolam has been well studied in terms of the hepatic and intestine metabolism [21]. Alprazolam is metabolized by CYP3A4 and CYP3A5 in the human liver [21],[22]. While CYP3A4 metabolizes alprazolam preferentially to 4-OHALP, metabolism mediated by CYP3A5 leads to the formation of both α- and 4-OHALP. Thus, the studies using human liver microsomes and recombinant CYP3A4 and CYP3A5 have shown that these two CYP enzymes are the major players in alprazolam metabolism. However, it has been recently reported that 65–85% of Asians, 70% of Caucasians and 27–50% of African Americans carry the CYP3A5*3 allele, which results in premature truncation and loss of enzyme activity. Thus, majority of individuals carrying this allele would be unable to metabolize ALP to α-OHALP [23].

CYP3A43 is a recently discovered P450 enzyme and has 75.7% sequence identity with CYP3A4 and 75.6% with CYP3A5 [16]. CYP3A43 gene is upstream of CYP3A4 and is opposite in orientation from all other 3A genes [16]. The expression of CYP3A43 in human liver is 0.1% that of CYP3A4 and 2% that of CYP3A5 [17]. Thus, the expression of CYP3A43 is relatively very low in liver, lung, and kidney and therefore, it has been concluded that CYP3A43 may not be a significant contributor to drug metabolism in humans [17].

Earlier studies from our laboratory had shown that relatively larger amount of α-OHALP is formed in human brain microsomes [4]. We, therefore, examined the relative expression of CYP3A4, CYP3A5, CYP3A7, and CYP3A43 in human brain cortex. Interestingly, we found that the relative expression of CYP3A43 was significantly higher in brain compared to the liver from the same individual (Fig 3b). In the 3 samples analyzed, CYP3A43 expression was 0.1% of CYP3A4 in liver while its expression was comparable to CYP3A4 in human brain cortex (Fig 3b). When we analyzed the relative expression of CYP3A43 and CYP3A4 in a larger set of 40 human brain autopsy tissues from the Indian population, we found that unlike the liver [16], CYP3A43 and CYP3A4 were expressed in equal amounts. In fact, in 3 out of 40 samples the expression of CYP3A43 was several fold higher than that of CYP3A4 (Fig 4a). In the 10 Caucasian samples analyzed CYP3A43 expression was substantially higher than CYP3A4 (Fig. 4c and d).

We also analyzed the relative expression level of all the four 3A genes in six brain samples and found that the expression of CYP3A5 was significantly less than CYP3A4 and CYP3A43 in the brain. Thus, its contribution to the generation of α-OHALP in brain would be less than that of CYP3A43. While earlier studies on the expression of CYP3A43 in human tissues have been done in a semi-quantitative fashion, we provide the first quantitative evidence for the expression of the CYP3A genes in human brain. It is plausible that autolytic changes during the postmortem interval could have contributed to this differential expression; however, our earlier studies [24] have shown the stability of brain RNA for up to 12 hr of postmortem interval.

Metabolism of ALP to its hydroxylated metabolites is often indicative of reduced bioavailability of the parent drug since the hydroxylated metabolites are unlikely to cross the blood brain barrier. However, if these pharmacologically active metabolites were to be generated *in situ* in the brain, they would exert therapeutic action and potentially have a longer half life due to the reduced clearance of the hydrophilic metabolites from the brain. The reported relative potencies in benzodiazepine receptor binding experiments and in animal models of seizure inhibition are 0.20 and 0.66, respectively, for 4-OHALP and α-OHALP. It is apparent that the α-OHALP is 3 times more potent that 4-OHALP and therefore if generated in the brain it would exert considerable therapeutic action.

We cloned and expressed recombinant CYP3A4 and CYP3A43 in COS-1 cells and examined the metabolism of ALP, in vitro. ALP was metabolized predominantly to 4-OHALP by recombinant CYP3A4, while recombinant CYP3A43 metabolized ALP to both α and 4-OHALP in equal amounts (Fig 5c). In the present study, we demonstrate the relatively higher expression of CYP3A43 in human brain and also the ability of recombinant CYP3A43 to metabolize ALP to both 4-OHALP and α-OHALP.

This is the first demonstration of the functional significance of CYP3A43 in terms of its ability to metabolize xenobiotics including drugs, although a weak testosterone hydroxylation activity has been demonstrated earlier [16]. However, the presence of CYP3A43 protein in human brain is yet to be demonstrated. The difficulty in generating antibodies to specific CYP3A enzymes has been a limiting factor since these enzymes show a high degree of sequence identity.

Domanski *et al* [16] had suggested that a high frequency of alternatively spliced forms of CYP3A43 may be produced and indeed, we have detected at least four alterative spliced forms in the human brain (Fig 8), of these, three have premature termination codon. One of them (having deletion of both the 7 and 8 exons and an insertion of 121bp of intron 7; Fig 8e) would generate a complete open reading frame. This is currently being characterized. The contribution of some of these spliced forms, which are expressed as functional proteins need to be examined.

The CYP3A genes are differentially expressed in the brain but they are also localized in distinct cell population and this would further contribute to differential drug metabolism. For example, CYP3A4 mRNA is predominantly localized in the Purkinje cell layer (Fig 2) in the cerebellum while CYP3A43 expression is completely lacking in this cell layer [4].

The presence of a relatively higher amount of CYP3A43 in human brain may also contribute to the metabolism of other psychoactive drugs that are metabolized by CYP3A enzymes. While it is acknowledged that the measurement of gene expression in the brain is relatively difficult and can only be done in autopsy tissue, it would be helpful to incorporate the study of CYP3A43 mediated metabolism using the recombinant enzyme. This would be particularly be useful in new drug development and would provide a measure of the relative contribution of the parent drug and the metabolites *in situ* in the target organ. It is to be seen whether the differential expression of CYP3A enzyme observed in the present study also extends to other members of the P450 family and if indeed it is so; their contribution to the metabolism of psychoactive drugs would be important determinants of the pharmacodynamics of drug action in the brain.

## Materials and Methods

### Human Tissue

Human tissues were obtained from male and female traffic accident victims with no known neurological or psychiatric disorders through the Human Brain Tissue Repository, NIMHANS, India, in compliance with the ethical guidelines of the Government of India. Autopsy was performed after obtaining informed consent from the next of kin and the protocol for use of autopsy tissue was cleared by the IRB of National Institute of Mental Health and Neurosciences, Bangalore and National Brain Research Centre, Manesar. For some of the experiments we also obtained brain tissue from normal control subjects at autopsy from the Washington DC and Northern Virginia Medical Examiners' Offices (from the Section on Neuropathology Clinical Brain Disorders Branch, GCAP, IRP, NIMH, NIH). Tissue was donated with informed consent from the next of kin under NIMH protocol. The average age of individuals was 38.2±18.9 (7 to 72 years) and postmortem delay between death and autopsy was 5.50±2.45 hr. After autopsy, the brains were washed in ice-cold saline and dissected into different regions based on standard anatomical markings. Tissues were frozen and stored at −70°C immediately. The tissues were thawed on ice prior to use. It was homogenized in 10 volumes of homogenization buffer consisting of 0.1M, pH 7.4 Tris-HCl (0.1M, pH 7.4) containing DTT (1 mM), KCl (1.15% v/v), PMSF (0.1 mM), BHT (22 mM), aprotinin (0.001% w/v), leupeptin (0.001% w/v) and glycerol (20% v/v). The buffer was bubbled with nitrogen before use. The homogenate was centrifuged at 100,000g for 1.5hr and the pellet was resuspended in minimum amount of homogenization buffer, aliquoted, snap frozen in liquid nitrogen and stored at −70°C until use.

### RT-PCR amplification, cloning, and localization

Total RNA was isolated from samples of human brain cortex using TRI reagent [25]. A region of the 3′UTR of CYP3A43 consisting of 85bp [26] was amplified by RT-PCR using total RNA from human brain. The amplicon was cloned and used to generate sense and antisense riboprobes for northern blot analysis and fluorescent *in situ* hybridization (FISH) experiments using T7 and T3 RNA polymerases, respectively. The complete open reading frame (ORF) and partial exon-specific regions of CYP3A4 and CYP3A43 were also amplified using RT-PCR. All the PCR products were verified by sequencing.

### Northern blotting

Total RNA was separated electrophoretically and transferred to a positively charged nylon membrane by capillary transfer [27], UV cross-linked and hybridised with digoxigenin labelled antisense riboprobes of the 3′UTR of CYP3A43 prepared using T7 and T3 RNA polymerases for generating sense and antisense riboprobes respectively. The membrane was hybridised overnight with digoxigenin labelled riboprobes at 52°C, washed, incubated with antibody to digoxigenin Fab fragments conjugated with alkaline phosphatase. The bands were visualized using a chromogenic substrate for alkaline phosphatase.

### Fluorescence *in-situ* hybridisation

Regions from human brain (cortex, cerebellum, midbrain and hippocampus) were dissected out and fixed in buffered paraformaldehyde. The tissue was processed for paraffin embedding and serial sections (8–10 µm thick) were cut under RNase-free conditions. Sections were dewaxed, hydrated in graded ethanol; acetylated and treated with proteinase K. The sections were rinsed in phosphate-buffered saline (PBS) and dehydrated using graded ethanol. Digoxigenin-labelled sense (for control sections) and antisense cRNA probes were synthesized from cDNA to CYP3A43-3′ UTR using T3 and T7 RNA polymerases. Sections were hybridised overnight at 55°C with the sense and antisense probes. After hybridisation, the sections were washed, blocked with 0.5% (w/v) bovine serum albumin and incubated with antibody to digoxigenin Fab fragments conjugated to horseradish peroxidase. After washing, the sections were incubated with biotinylated tyramide (NEN Life sciences Products, USA) followed by FITC labelled streptavidin. Finally the sections were washed, dried and mounted prior to examination under a fluorescence microscope.

### Quantitative real-time PCR

The relative expression of CYP3A4, CYP3A5, CYP3A7, and CYP3A43 was quantitated in human brain cortex by real-time PCR using the primer sets reported by Williams et al. 2004 [26]. iQ SYBR green Supermix from Biorad was used. The conditions for real-time PCR were as follows: 95°C for 3 min (1 cycle), 94°C for 20 sec, 62°C for 30 sec, and 72°C for 30 sec (40 cycles), 72°C for 3 min (1cycle). The real-time PCR results were analyzed using the iCycler Thermal Cycler software (Biorad). qRT-PCR for Caucasian-American Samples were done using an ABI Prism 7700 sequence detection system (Applied Biosystems, Foster City, CA) housed in the Quantitative Genomics Lab Core facility at the University of Texas Health Science Center, Houston. Amplification for each assay was carried out at 95°C for 1 minute, followed by 40 cycles of a 12 second step at 95°C and a 30 second step at 60°C. Taqman® style primer and probe sets were designed to overlap the exon/exon junctions of mRNA using the Primer Express software (Applied Biosystems). The probes contain a 5′ 6-FAM (5-carboxyfluorescein) and a 3′ TAMRA (5-carboxytetramethylrhodamine). The sequences for the primer and probe sets used for the CYP3A4 and CYP3A43 assays can be found in the previous publication by Williams et al. 2004 [26]. All reactions were normalized with 18S rRNA as internal control and all reactions were carried out in triplicates.

### Experiments with recombinant CYP3A4 and CYP3A43

Complete ORFs of CYP3A4 and CYP3A43 were cloned into mammalian expression vector and transiently transfected into COS-1 cells. Cells transfected with vector alone were used as control. After antibiotic selection, cells were harvested after 48hrs in cold DMEM media. The cells were centrifuged at 1500g for 10 min at 4°C. The cell pellet was washed twice with the homogenization buffer consisting of Tris-HCl (0.1M, pH 7.4) containing DTT (1 mM), KCl (1.15% v/v), PMSF (0.1 mM), BHT (22 mM), aprotinin (0.001% w/v), leupeptin (0.001% w/v) and glycerol (20% v/v). The buffer was bubbled with nitrogen before use. Finally, the pellet was resuspended in minimum amount of homogenization buffer and homogenized using Potter-Elvehjem homogenizer with 20 up and down strokes. The cell homogenate prepared as above was aliquoted and kept frozen at −70°C and used to study the metabolism of alprazolam. For immunoblotting, antibody to CYP3A was procured from BD Gentest; NJ, USA was used.

### Metabolism of Alprazolam

Human brain total membrane preparations (containing microsomal and mitochondrial protein) from human brain cortex containing microsomal and mitochondrial protein and total lysates from cells expressing recombinant CYP3A4 and CYP3A43 cells preparations (1 mg/ml protein) were preincubated with alprazolam (1 mM) at 37°C for 4 min. The reactions were initiated by addition of NADPH (5 mM) and purified liver NADPH-cytochrome P450 reductase (1 unit). The reactions were terminated after 30 min by the addition of NH_4_OH (0.1%, v/v). The incubations were extracted thrice by using dichloromethane (2 ml), vortexed, centrifuged at 4000 g (5 min) and the lower organic phase was pooled and evaporated under nitrogen gas at 45°C. The residue was dissolved in 0.4 ml of the HPLC mobile phase consisting of potassium phosphate buffer (50 mM, pH 9.0) containing acetonitrile (30% v/v) filtered and injected into an HPLC column consisting of (SUPELCOSIL, (LC-C18 column) at the flow rate of 1 ml per min^−1^. Blank reactions did not contain NADPH. Formations of metabolites were detected using a UV detector set at 224 nm. Standards 4-hydroxy alprazolam, α-hydroxy alprazolam, and alprazolam were used for quantification of metabolites.
